# Properties of Myofibrillar Protein in Frozen Pork Improved through pH-Shifting Treatments: The Impact of Magnetic Field

**DOI:** 10.3390/foods13131988

**Published:** 2024-06-24

**Authors:** Bo Chen, Gaoang Du, Ke Li, Yu Wang, Panpan Shi, Junguang Li, Yanhong Bai

**Affiliations:** 1 College of Food and Bioengineering, Zhengzhou University of Light Industry, Zhengzhou 450001, China; chenboemail163@163.com (B.C.);; 2 Key Laboratory of Cold Chain Food Processing and Safety Control, Ministry of Education, Zhengzhou 450001, China

**Keywords:** frozen meat, myofibrillar protein, pH-shifting, magnetic field, protein modification

## Abstract

The present study demonstrates the effects of pH-shifting treatments and magnetic field-assisted pH-shifting treatments on the properties of myofibrillar protein (MP) in frozen meat. The solubility results indicate that the pH-shifting treatments increased the solubility of MP from 16.8% to a maximum of 21.0% (pH 9). The values of surface hydrophobicity and protein particle size distribution indicate that the pH-shifting treatment effectively inhibited protein aggregation through electrostatic interactions. However, under higher pH conditions (pH 10, 11), the treatments assisted by the magnetic field increased the degree of aggregation. The total thiol content and SDS-PAGE results further suggest that the magnetic field-assisted pH-shifting treatment accelerated the formation of covalent bonds among MPs under the alkaline environment. The results of the Differential Scanning Calorimetry (DSC) and protein secondary structure analysis indicate that the magnetic field promoted the unfolding of protein structures in an alkaline environment, markedly reducing the effective pH levels of pH-shifting. Electron paramagnetic resonance (EPR) data indicate that the phenomenon might be associated with the increased concentration of free radicals caused by the magnetic field treatment. In summary, the application of magnetic field-assisted pH-shifting treatments could emerge as a potent and promising strategy to improve the protein properties in frozen meat.

## 1. Introduction

Freezing is the predominant method for meat product preservation. However, long-term frozen storage usually leads to increased drip loss and reduced meat quality. This is primarily attributed to the high proportion of water being converted into ice crystals which are then trapped within the myofibrils; moreover, the deterioration in quality is especially severe during a slow freezing process due to the formation of larger ice crystals within the muscle fibers, which accelerates protein denaturation [1]. In response, several innovative rapid freezing and thawing techniques have been introduced. These include ultrasonic-assisted, ultra-high pressure-assisted, electric field-assisted freezing, and magnetic field-assisted thawing. Such methods aim to reduce the muscle damage caused by ice crystallization, thereby improving the overall quality of meat processing [2].

However, the ice crystals during the frozen storage inevitably damage meat tissue and leads to protein denaturation. This challenge in the quality improvement of frozen meat may be addressed with reference to improving the processing properties of PSE (pale, soft, and exudative)/wooden meat, where the proteins undergo denaturation thus affecting their processing characteristics [3]. Lu et al. [4] utilized the pH-shifting treatment to process the MPs obtained from PSE-like chicken breast for improving the protein gelling and emulsifying properties. Wang et al. [5] reported that the pH-shifting process prompted the unfolding of MPs obtained from chicken wooden breast, which was conducive to the interaction among proteins or between proteins and water. Therefore, it appears feasible to use pH-shifting techniques to improve the quality of frozen meat. However, its practical application still faces some challenges, primarily because higher pH values are necessary for effective protein modification. In the study of Lu et al. [4], the emulsion stabilized by MPs was subjected to treatment under pH 11.0 or pH 11.5 and exhibited uniform and dispersed particle distributions with little aggregation. Pezeshk et al. [6] obtained optimal modification of shrimp by-products at a pH of 12.5. Li et al. [7] indicated that treatment at pH 12 significantly improved the solubility and emulsification of myosin compared to pH 9, 10, and 11. Such an increase in pH values, on the one hand, contradicts green processing principles, and on the other hand, it could facilitate the formation of covalent bonds among MPs, leading to protein aggregation [4].

Recent studies have indicated that combining pH-shifting treatments with physical methods can enhance the effectiveness of the pH-shifting process [8]. Due to their less stable structure, proteins in base environments tend to be more responsive to treatments such as heat, ultrasound, and high hydrostatic pressure. Wang et al. [5] introduced the high-intensity ultrasound (HIU) into the unfolding–refolding process of pH-shifting treatment and reported that the functional properties of MPs could only be improved by the assistance of HIU during the unfolding phase of the pH-shifting. Chang et al. [9] investigated the effects of temperature on the properties of a pea protein modified by pH-shifting. The results demonstrated that a simple pH shift combined with a controlled heating technique could be applied to improve the foaming properties of the pea protein. Magnetic fields are often used as a non-polluting, user-friendly, and environmentally sustainable treatment method. Previous studies have shown that magnetic fields can alter the hydrogen bond content in water, potentially weakening or disrupting the hydrogen bond network within water clusters. This leads to the fragmentation of larger water clusters into smaller, more stable ones, releasing free water molecules [10]. This unique property of magnetic fields has been utilized in the freezing process of meat to inhibit the phase transition of water molecules, thereby decelerating ice recrystallization during freezing [11]. The characteristic of pH-shifting is to charge the amino acids in the protein, which then interact with water molecules through ion–dipole interactions, increasing the solubility of the protein. Concurrently, proteins with identical charges repel each other, resulting in the dissociation of subunits and the unfolding of protein structure. Inspired by this, the study proposed the incorporation of the magnetic field into the pH-shifting process for treating MP, aimed at providing a new way to improve the processing characteristics of frozen meat and explore the effectiveness of magnetic fields to enhance pH-shifting effects.

## 2. Materials and Methods

### 2.1. Materials

The pork *longissimus thoracis et lumborum* muscle (Landrace × Yorkshire × Duroc pigs aged six months, male, 200 kg, 24 h post-mortem) was obtained from a local supermarket (Zhengzhou, Henan, China) and immediately transported to the laboratory at 4 °C. The samples were trimmed to remove the fat and connective tissue. Then, the samples were split into bulk samples of 100 g (15 cm × 10 cm × 5 cm) and packed in vacuum-sealed bags. The samples were stored at −20 ± 1 °C for three months before the experiments. NaCl, NaOH, HCl, MgCl_2_, EGTA (ethylene-bis (oxyethylenenitrilo) tetraacetic acid), PIPES (1,4-piperazinediethanesulfonic acid), and phosphates were purchased from the Sinopharm Chemical Reagent Co., Ltd. (Shanghai, China), and other chemicals and reagents were at least of analytical grade.

### 2.2. Preparation of Samples

The frozen meat was thawed completely at 4 °C, minced with a grinder, and then mixed with four volumes of the buffer (10 mmol/L Na_2_HPO_4_, NaH_2_PO_4_, 0.1 mol/L NaCl, pH 6.5). Next, the minced meat was separated into the pH-shifting treatment and the magnetic field-assisted pH-shifting groups. In the pH-shifting treatment groups, the pH of the mixtures was adjusted to 9.0, 10.0, and 11.0, using 2 mol/L NaOH, respectively, and equilibrated at 4 °C for 20 min thereafter. In the magnetic field-assisted pH-shifting treatment groups, the mixtures were first adjusted to the required pH and subsequently transferred into the magnetic field test chamber (MIF-FI, Indust, Wuxi, China) for the magnetic field treatment. The intensity of the magnetic field was set to 3 mT and the treatment time was 20 min. Afterward, all the mixtures were neutralized to pH 6.5, using 2 mol/L HCl, and maintained at 4 °C for 10 min. The control group was only mixed with four volumes of buffer without any other treatments. Finally, the treatment groups and control group were centrifuged at 600× *g* (Avanti J-26S XPI, Beckman Coulter, Brea, CA, USA) for 15 min (4 °C).

The method of extracting MPs from the prepared meat samples was adopted from the study by Guo et al. [12]. The processed minced meat was mixed with four volumes of extracting buffer (10 mmol/L Na_2_HPO_4_/NaH_2_PO_4_, 0.1 mol/L NaCl, 2 mmol/L MgCl_2_, 1 mmol/L EGTA, pH 7.0) and homogenized at 10,000× *g* for 60 s using the high-speed homogenizer (Ultraturrax T25, IKA Works GmbH & Co., Staufen, Germany). The homogenate was then centrifuged at 2000× *g* for 15 min (Avanti J-26S XPI, Beckman Coulter, Brea, CA, USA) and the supernatant was discarded. The pellet was washed twice more with 4 volumes of the above buffer under the same centrifugation conditions. Subsequently, the myofibril pellet obtained was washed three times with 4 volumes of 0.1 mol/L NaCl. The MPs were diluted with the buffer (0.6 mol/L NaCl, 25 mmol/L PIPES, pH 6.5), and the MP concentration was measured using the Biuret method [13]. At last, the MP samples were stored at 4 °C and used within 72 h.

### 2.3. Determination of Protein Solubility

The solubility of the MPs was determined as follows: 10 mL of MP dispersion (5 mg/mL) was centrifuged at 10,000× *g* for 20 min. Then, the supernatant was collected and the protein content was measured using the Bradford method [14]. Protein solubility is expressed as the percentage of protein concentration before and after centrifugation [13].

### 2.4. Particle Analysis

The particle size distribution of protein samples was determined by dynamic light scattering (DLS) (Zetasizer Nano-ZS 90, Malvern Instruments, Malvern, UK) using a slight modification of the protocol proposed by Cheng et al. [15]. Briefly, the MP dispersion with different treatments were prepared at a concentration of 0.5 mg/mL, and 1.2 mL aliquots of the protein suspension were transferred into quartz cuvettes with a 1 cm optical path length. The samples were then equilibrated for 180 s inside the instrument, and the data were collected over 12 sequential readings at a scattering angle of 90°.

### 2.5. Determination of Total Sulfhydryl Content

The SH content was measured according to the method of Han et al. [16]. Briefly, the MP samples (0.5 mL, 1.0 mg/mL) were dissolved in 4.5 mL of solubilizing buffer, consisting of 0.2 mol/L Tris-HCl, 8.0 mol/L urea, 1.0% SDS, and 3.0 mmol/L EDTA. Subsequently, a mixture of 4 mL of the supernatant and Ellman’s reagent (0.5 mL, 2.0 mmol/L DTNB, 0.2 mol/L Tris-HCl, pH 8.0) was then incubated at 40 °C for 25 min. A molar extinction coefficient of 13,600 M^−1^·cm^−1^ was used to calculate the SH content from the absorbance measured at 412 nm.

### 2.6. Determination of Surface Hydrophobicity

The protein was resuspended in the buffer (0.6 mol/L NaCl, 25 mmol/L PIPES, pH 6.5) to obtain a concentration of 5 mg/mL, and the surface hydrophobicity was determined in accordance with the procedure described by Li et al. [17]. A protein sample (4 mL) was mixed with 80 mL of bromophenol blue (BPB) and incubated at 20 °C for 10 min, followed by centrifugation at 2000× *g* for 15 min to collect the supernatant. After that, the absorbance of the supernatant was measured at 595 nm against a phosphate buffer blank. An index of hydrophobicity was determined by the amount of BPB bound, given by the following equation:

BPB bound (μg) = 200 μg × (OD control − OD sample)/OD control

### 2.7. SDS-PAGE

Using the method of Bai et al. [18], the SDS-PAGE pattern changes of the MPs were determined under reducing and non-reducing conditions. Under reducing conditions, the MP dispersion (1 mg/mL) was mixed in a 1:1 (*v*:*v*) ratio with the buffer (0.5 mol/L Tris-HCl, 20% (*v*/*v*) Glycerol, 10% (*w*/*v*) SDS, 3.33% (*w*/*v*) DTT, and 2% (*w*/*v*) bromophenol blue). The mixture was then heated in a boiling water bath at 100 °C for 5 min. Under non-reducing conditions, to prevent disulfide artifact formation during boiling, protein samples without DTT were treated with 1 mmol/L N-ethylmaleimide (a thiol-blocking agent). After centrifugation at 10,000× *g* for 5 min, 10 µL of the protein suspension samples were loaded into polyacrylamide gels composed of a 10% separating gel and a 4% stacking gel for electrophoresis (Bio-Rad Mini-PROTEANetra electrophoresis system). Following electrophoresis at 80 V for the stacking gel and 120 V for the separating gel, the gel was stained for 1 h in Coomassie Brilliant Blue R-250 and then destained in 8% acetic acid and 25% ethanol for 12 h. A pre-stained marker ranging from 11 to 245 kDa was used to indicate the molecular weight of the relevant protein bands in the protein gels.

### 2.8. DSC Analysis

A DSC-Q200 (TA, New Castle, DE, USA) was used to evaluate the thermal characteristics of MP samples for a temperature range of 25 to 100 °C, following the method reported by Zhang et al. [19]. A total of 20 mg of protein samples were precisely measured and sealed in aluminum pans for heating, with an empty sealed pan used as a thermal reference. The peak temperatures (T) and enthalpy values (ΔH) were obtained from the DSC thermograms.

### 2.9. Determination of Protein Secondary Structure

In accordance with Zhao et al. [20], the secondary structure of the protein samples was determined using a Chirascan-qCD spectropolarimeter at 25 °C. Far-UV CD spectra of 0.2 mg/mL protein suspensions were recorded from 200 to 250 nm. All samples were scanned three times at a speed of 60 nm/min with 0.5 nm step intervals. The CD spectrum is composed of the average data from three scans. The residue elliptic values [θ] in deg cm^2^ dmol^−1^ are displayed as the CD data.

### 2.10. Amino Acid Composition Analysis

The amino acid content of the protein samples was determined according to Liu et al. [21], with some modifications, using a Hitachi automatic amino acid analyzer (L-8900). Briefly, the protein samples (10 mL) were hydrolyzed with 6.0 mol/L HCl in a closed digestion tube and subsequently kept in an oven at 110 °C for 24 h. After hydrolysis, the tubes were removed, cooled, and the dried substance was dissolved with 0.02 mol/L HCl. The solution was transferred to a 50 mL volumetric flask and adjusted to 50 mL with deionized water. A portion of the solution was then transferred to a centrifuge tube, centrifuged at 10,000× *g* for 5 min, and the supernatant was filtered through a 0.22 μm membrane. The wavelength of the detector was 254 nm. Standard curves were generated using commercially available amino acid mixtures.

### 2.11. Electron Paramagnetic Resonance (EPR) Measurements

The redox status of the meat samples under the pH 11 treatment was investigated using the spin-trapping EPR method proposed by De Zawadzki et al. [22]. Firstly, 100 μL of DMPO was added to 2 mL of meat homogenate. The mixture was then treated at pH 11 with or without the magnetic field. After that, the samples were centrifuged for 10 min at 12,000× *g* to collect the supernatant. Aliquots of the supernatant (1.0 mL) were incubated for over 2 h in the 65 °C water bath before the EPR spectra were recorded. After cooling, EPR measurements were performed using an EPR EMX-Plus spectrometer (Bruker BioSpin, Rheinstetten, Germany). The following instrument settings were used for sample acquisition: microwave power of 10 mW; modulation frequency of 100 kHz; modulation amplitude of 1.0 G; magnetic field scan of 100 G; sweep time of 168 s; and sample temperature of 25 °C.

### 2.12. Statistical Analysis

Three independent tests were conducted with three batches of MP samples. For each batch of protein samples, the data were expressed as mean ± standard deviation. To identify the treatment effects and determine the differences between the individual means, the SPSS 19.0 statistical software (SPSS Inc., Chicago, IL, USA) was used for the analysis of variances with a significance level of *p* < 0.05 (*Tukey’s test*).

## 3. Results and Discussion

### 3.1. Solubility and Particle Size

**Figure 1:**
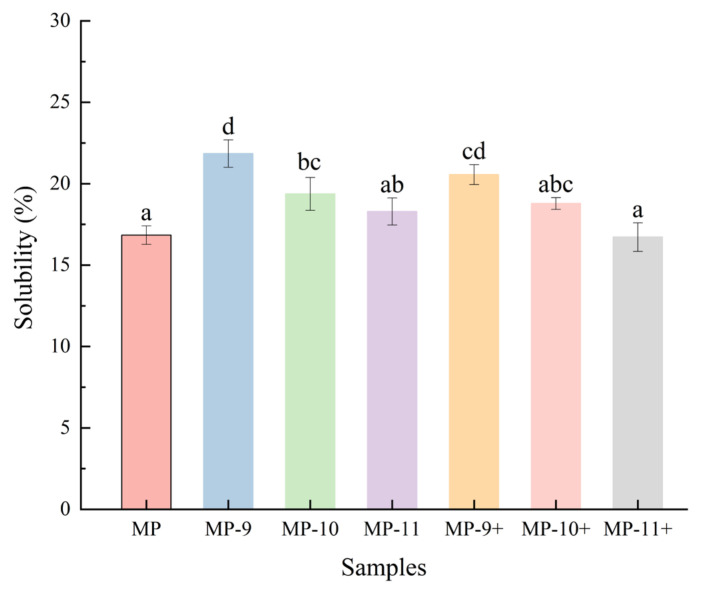
The effect of different pH-shifting treatments on the solubility of MPs. Values are given as the means ± SD from triplicate determinations. Different letters represent significant difference (*p* < 0.05). Note: MP represents the untreated sample. MP-9, MP-10, MP-11 represent the samples of the pH-shifting treatments to pH 9, pH 10, pH 11. MP-9+, MP-10+, and MP-11+ represent the samples subjected to magnetic field-assisted pH-shifting treatments, achieving pH levels of 9, 10, and 11, respectively.

Solubility significantly influences the functional properties of MP, including emulsification and gelation [13]. Consequently, a decrease in MP solubility is commonly used as the primary indicator for assessing the protein modifications resulting from freezing [23]. As shown in Figure 1, the solubility of MPs in frozen meat was initially 16.8%. After the pH-shifting treatments, the MP solubility improved, with the highest value being 21.9% for MP-9. Nevertheless, the further increase in pH reduced MP solubility, reaching 18.2% at pH 11, a value not significantly different from that of the control group (*p* > 0.05). In the magnetic field-assisted pH-shifting groups, the trend of the MP solubility changes aligned with the prior groups, showing a further reduced solubility at pH 11 (16.7%). During the freezing process, the MPs were damaged by the formation of ice crystals and an increase in ionic strength in the unfrozen water, leading to protein denaturation and aggregation [24], as indicated by a decrease in hydrogen bonds coupled with increased surface hydrophobicity. The enhancement of protein solubility by the pH-shifting treatments was attributed to the increased surface charge of the protein with the increasing pH, which increased the electrostatic force between protein molecules and their interaction with water molecules [25]. Nevertheless, the continued rise of pH levels (pH 10, pH 11) decreased protein solubility. This phenomenon is typically considered to be due to the fact that the hydrophobic forces, which usually contribute significantly to protein stability, become insufficient to counterbalance the electrostatic free energy generated at extremely lower or higher pH levels. Upon unfolding, hydrophobic groups within the protein become exposed, and the electrostatic free energy of the charged protein decreases. This reduction in electrostatic free energy occurs because the charge repulsion diminishes as the charges are distributed over a larger volume in the unfolded protein. [26]. Additionally, the thiol groups within the MPs were prone to oxidation to form disulfide bonds in alkaline conditions [24]. The intensified hydrophobic interactions and potential disulfide bonds among the protein molecules triggered the aggregation, thereby diminishing protein solubility.

**Figure 2:**
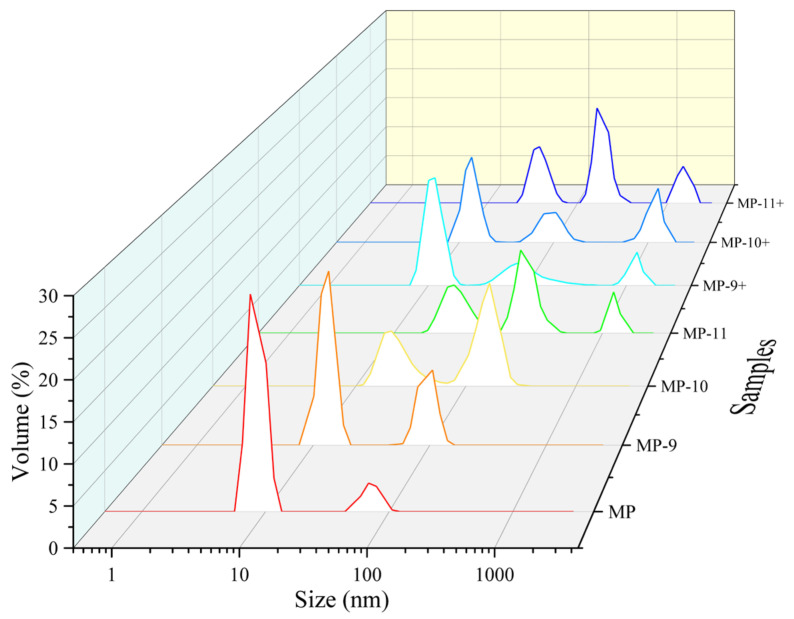
The effect of different pH-shifting treatments on the particle size distribution of MPs. Note: MP represents the untreated sample. MP-9, MP-10, MP-11 represent the samples of the pH-shifting treatments to pH 9, pH 10, pH 11. MP-9+, MP-10+, and MP-11+ represent the samples subjected to magnetic field-assisted pH-shifting treatments, achieving pH levels of 9, 10, and 11, respectively.

Figure 2 illustrates the protein particle size distribution of the samples, indicating that the MPs subjected to pH-shifting exhibit an increasingly broader particle size distribution, and the elevated pH levels correspondingly result in enlarged protein particle sizes. In the magnetic field-assisted pH-shifting groups, the protein size distribution shifts from bimodal to trimodal, consistently featuring a third peak over 1000 nm. This implies that the application of the magnetic field enhanced the reactions among the MPs during the pH-shifting treatment process.

### 3.2. Surface Hydrophobicity

**Figure 3:**
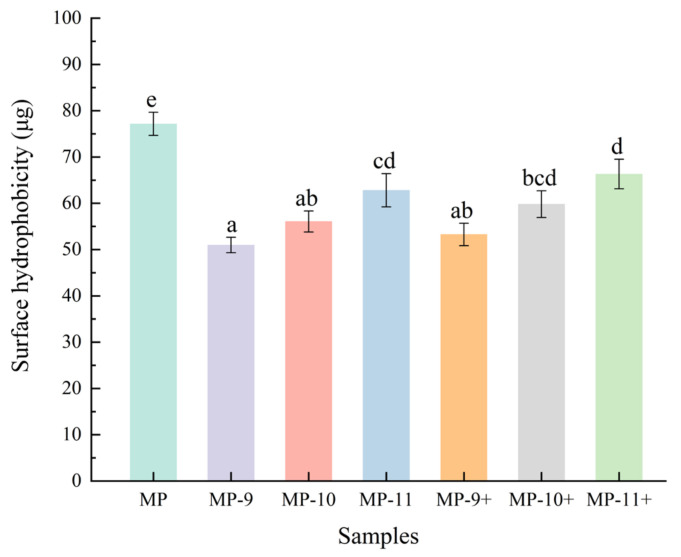
The effect of different pH-shifting treatments on the surface hydrophobicity of MPs. Values are given as the means ± SD from triplicate determinations. Different letters represent significant difference (*p* < 0.05). Note: MP represents the untreated sample. MP-9, MP-10, MP-11 represent the samples of the pH-shifting treatments to pH 9, pH 10, pH 11. MP-9+, MP-10+, and MP-11+ represent the samples subjected to magnetic field-assisted pH-shifting treatments, achieving pH levels of 9, 10, and 11, respectively.

The surface hydrophobicity of the protein is indicative of the extent of exposed hydrophobic amino acid residues on their surface and is commonly linked to changes in the tertiary structures of the protein [27]. It is well established that protein hydrophobic cores become exposed during the freezing process [28]. Figure 3 demonstrates that the surface hydrophobicity of all the groups is markedly lower than that of the control, suggesting improved tertiary structures in the MPs due to increased electrostatic repulsion from the pH-shifting treatments. However, the surface hydrophobicity of the pH-shifted samples increased with rising pH levels. As previously reported, proteins unfold at extreme pH levels and attempt to refold in environments adjusted to neutral pH, but their tertiary structures will not be fully restored, resulting in the exposure of hydrophobic groups [25]. Compared to the pH-shifting treatments, the upward trend in surface hydrophobicity was more pronounced in the magnetic field-assisted pH-shifting groups, demonstrating a marked impact on protein unfolding induced by increased pH values. Additionally, the oxidation of sulfhydryl groups under higher alkaline conditions further increased protein denaturation, thereby facilitating an enhancement in hydrophobicity.

### 3.3. Total Sulfhydryl Content

**Figure 4:**
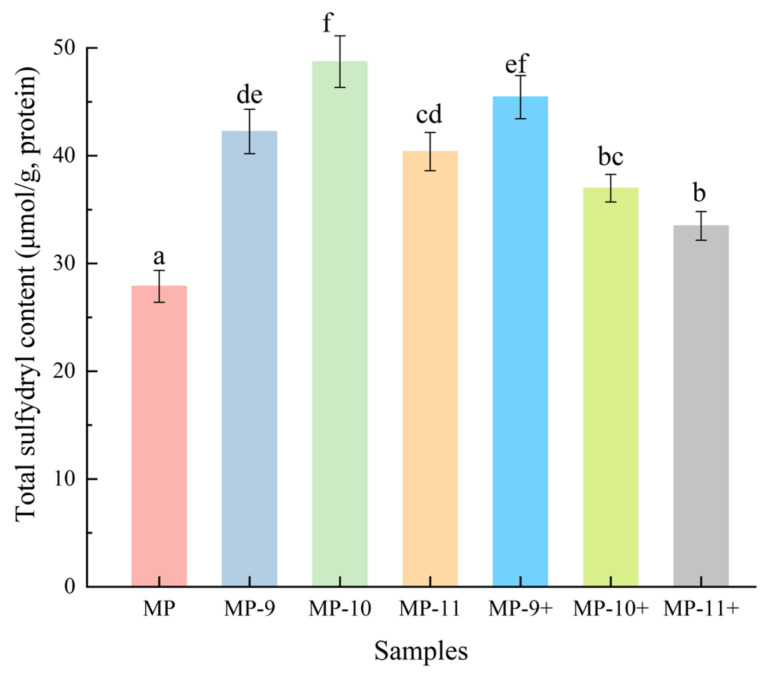
The effect of different pH-shifting treatments on the total sulfhydryl content of MPs. Values are given as the means ± SD from triplicate determinations. Different letters represent significant difference (*p* < 0.05). Note: MP represent the untreated sample. MP-9, MP-10, MP-11 represent the samples of pH-shifting treatments to pH 9, pH 10, pH 11. MP-9+, MP-10+, and MP-11+ represent the samples subjected to magnetic field-assisted pH-shifting treatments, achieving pH levels of 9, 10, and 11, respectively.

As shown in Figure 4, the sulfhydryl content of the control group is about 28 μmol/g, and the pH-shifting treatments significantly increase the sulfhydryl content in the MP samples. Specifically, the content levels in the MP-9, MP-9+, and MP-10 groups were elevated to around 42, 45, and 49 μmol/g, respectively, demonstrating marked increases of 50%, 60%, and 75% compared to the control. However, the sulfhydryl content did not significantly differ (*p* > 0.05) between the MP-9+ and MP-10 groups. Notably, in the MP-11, MP-10+, and MP-11+ groups, a downward trend of sulfhydryl content was observed, with the MP-10+ group showing no significant differences compared to that of MP-11+ (*p* > 0.05). Several studies have provided evidence supporting the reduction in SH groups and the formation of disulfide bonds during the prolonged frozen storage [29]. Furthermore, upon aggregation through hydrophobic interactions, some sulfhydryl groups located on the protein surface could be masked, consequently reducing the measurable sulfhydryl content in protein [30]. The hydrophobic interactions among the protein molecules were substantially decreased by the electrostatic interaction through the pH-shifting treatments, consequently mitigating aggregation and increasing the total sulfhydryl group content detectable within the protein. The noted decline in sulfhydryl content with further pH elevation suggests that in an alkaline environment, sulfhydryl groups begin to oxidize into disulfide bonds. The decreased sulfhydryl content in the magnetic field-assisted pH-shifting treatment groups, relative to those at the same pH level, implies that the magnetic field could accelerate the oxidation process of sulfhydryl groups within the system.

### 3.4. SDS-PAGE

**Figure 5:**
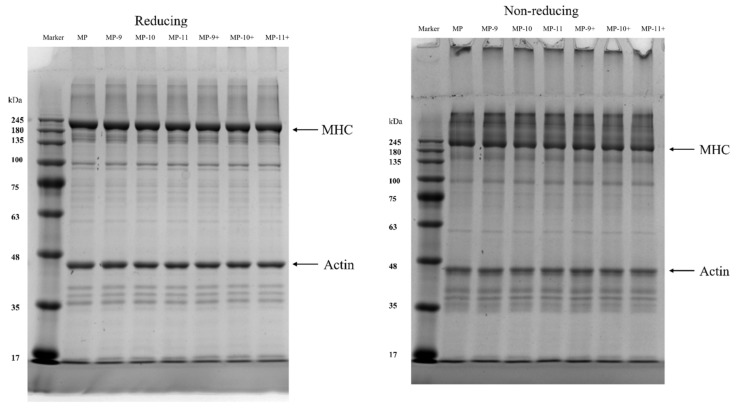
The effect of different pH-shifting treatments on the SDS-PAGE patterns of MPs. Note: MP represents the untreated sample. MP-9, MP-10, MP-11 represent the samples of pH-shifting treatments to pH 9, pH 10, pH 11. MP-9+, MP-10+, and MP-11+ represent the samples subjected to magnetic field-assisted pH-shifting treatments, achieving pH levels of 9, 10, and 11, respectively.

Both the reducing and non-reducing SDS-PAGE analyses were performed to further investigate the effects of the pH-shifting treatments on the formation of disulfide bonds in the MPs. It can be seen in Figure 5 that the MPs display two main bands near 245 kDa and 48 kDa, which correspond to the actin and myosin heavy chains, respectively. No significant differences among the band patterns of different samples were observed in the reducing SDS-PAGE. In contrast, a distinct band above the MHC in the non-reducing SDS-PAGE almost disappeared following the reducing SDS-PAGE, indicating that the band was predominantly composed of protein complexes formed via disulfide bonds. The results suggest that the protein complexes that remained above the MHC band under reducing conditions primarily consisted of non-disulfide covalent complexes induced by the alkaline environment [31]. In addition, in non-reducing electrophoresis, the intensity and width of the MHC bands gradually decreased with the increase in pH. This trend was particularly pronounced in the group subjected to the magnetic field-assisted pH-shifting treatment, where the MHC bands in MP-11+ showed a significantly decreased trend of protein content. In non-reducing electrophoresis, the protein aggregates formed by disulfide bonds remained intact due to the inability of the SDS buffer to dissociate them. Given their substantial molecular size, these aggregates tended to be precipitated during the centrifugation phase prior to electrophoretic loading or become entrapped in the upper segment of the electrophoretic gel, consequently reducing the protein concentration in the loaded samples.

### 3.5. Thermal Denaturation

The thermal denaturation temperatures of myosin (T_1_) and actin (T_2_) are listed in Table 1. As the pH increased, the values of T_1_ and T_2_ decreased, indicating that the alkaline environment destabilized the structure of MP. However, the changes in T_1_ were not as significant as those in T_2_. Notable variations in T_1_ occurred only in the MP-11+ group, whereas significant differences in T_2_ were observed starting from the MP-9 group, suggesting that actin was more susceptible to alkaline-induced damage than myosin. Enthalpy changes were associated with the disruption of internal interactions (e.g., hydrogen bonds, Van der Waals forces) within the protein molecules. When the proteins unfold, the hydration of the groups that were previously buried become exposed, leading to a decrease in enthalpy [32]. The significant decrease in enthalpy for both myosin and actin indicate that the alkaline environment significantly disrupted the tertiary structure of MP, in agreement with the results related to surface hydrophobicity. Under extreme alkaline conditions, the protein would only be partially unfolded, retaining a relatively compact state while losing most of its side-chain interactions (e.g., tertiary structure) [33]. The relatively intact secondary structure of myosin accounted for the smaller decline in its thermal denaturation temperature under moderately low pH conditions. In the group treated with magnetic field-assisted pH-shifting, a more rapid decrease in thermal denaturation temperature of myosin suggests that the magnetic field enhanced the alkaline-induced disruption of the protein structure.

### 3.6. Protein Secondary Structure

**Figure 6:**
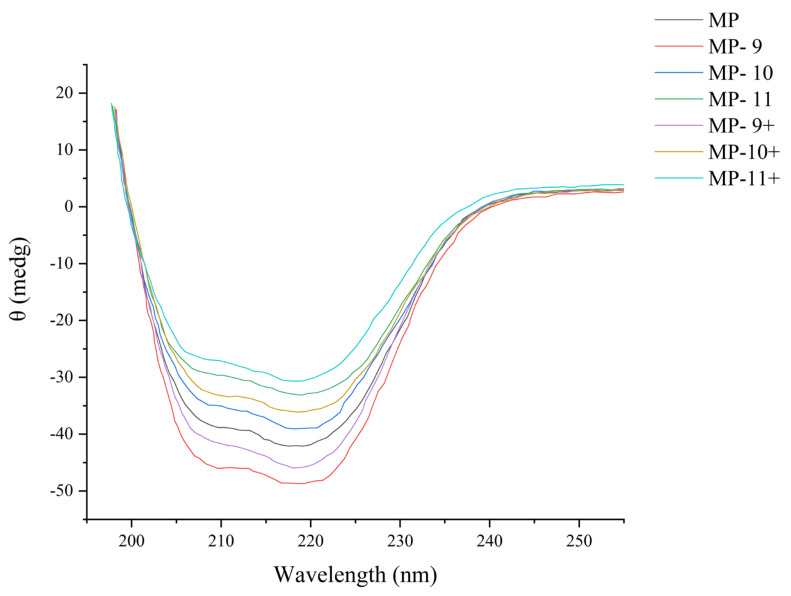
The effect of different pH-shifting treatments on the CD spectra of MPs. Note: MP represents the untreated sample. MP-9, MP-10, MP-11 represent the samples of pH-shifting treatments to pH 9, pH 10, pH 11. MP-9+, MP-10+, and MP-11+ represent the samples subjected to magnetic field-assisted pH-shifting treatments, achieving pH levels of 9, 10, and 11, respectively.

Figure 6 showed significant changes in the CD spectra of the MPs under the different pH-shifting treatments. The two peaks near 208 and 222 nm, characteristic of the α-helix structure, reached their maximum intensity in the MP-9 group, indicating a highly preserved secondary protein structure, followed by the MP-9+ group. A continued rise in pH led to a lower peak intensity of the α-helix peaks compared to the native protein, with the lowest intensity in the MP-11+ group, suggesting the pronounced loss of α-helix and the most severe damage of secondary structure among all the treated groups. Under freezing conditions, the protein structure was damaged and aggregated through hydrophobic interactions. The extreme pH values could disrupt hydrophobic interactions through the electrostatic forces, causing the protein structure to unfold. When pH was re-neutralized, the unfolded polypeptide chains refolded, partially refolding the secondary structure [25]. Due to the opposite polarity, the light chains primarily encircled a long α-helix in the neck region of the head through both polar and electrostatic interactions in their native state, and the heavy chain of myosin remained associated under alkaline conditions, whereas a fraction of the light chain dissociated. [34]. When the pH was readjusted to neutrality, the light chains remain dissociated, resulting in the loss of secondary structure in the head fraction of myosin. Progressively increasing pH levels caused more extensive protein unfolding and disorganized refolding. Hence, the pH-shifting treatment resulted in a more disordered secondary structure of MPs under elevated pH conditions compared to the native state. Observations from the magnetic field-assisted pH-shifting groups suggest that the magnetic field expedited the unfolding of light chains in alkaline conditions.

### 3.7. Amino Acid Profile

The amino acid composition of proteins is crucial to their nutritional value and functionality. Table 2 shows that the amino acid levels remained consistent across different treatments. Amino acids, such as arginine, lysine, and histidine, which are typically vulnerable to loss within oxidative chemical reactions, showed relative stability. This may be due to their basic nature, contributing to their stability in alkaline conditions. Similarly, acidic amino acids, including aspartic acid and glutamic acid, which were typically prone to deprotonation and deamidation under alkaline conditions, retained their contents, indicating that the pH treatments had minimal impact on the primary structure of MP. Consequently, the treatments appeared to preserve the nutritional value of meat products.

### 3.8. Analysis of EPR Results

**Figure 7:**
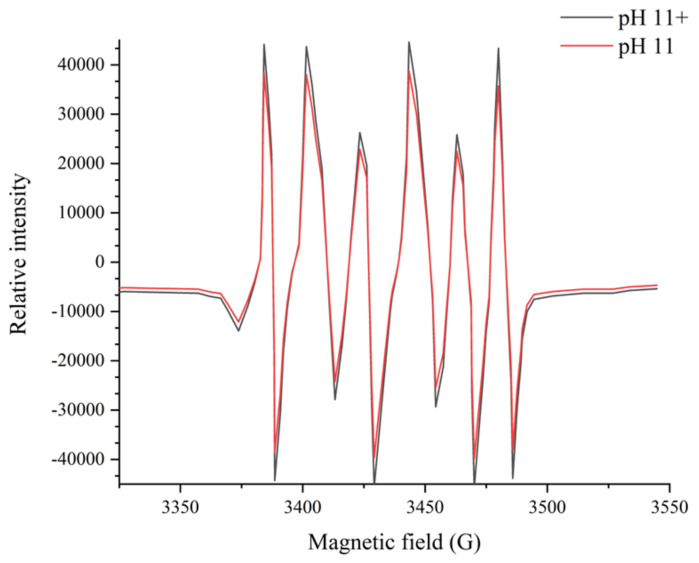
The EPR spectra of superoxide anions (O_2_^−^) obtained from the samples of the pH-shifting treatment (pH 11) and the magnetic field-assisted pH-shifting treatment (pH 11+).

**Figure 8:**
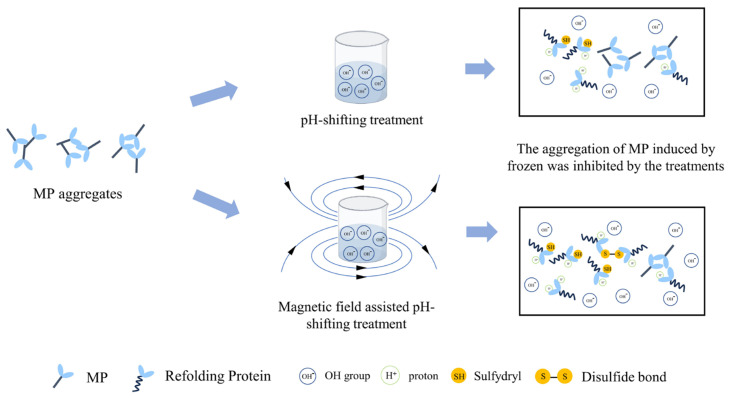
The schematic diagram of the influence of pH-shifting treatments and the magnetic field-assisted pH-shifting treatments on the MP properties.

To further clarify the impact of magnetic fields on pH-shifting treatments, the EPR results are depicted in Figure 7. The spin trap DMPO effectively captured hydroxyl radicals and superoxide anions, representing two distinct radical types. Hydroxyl radicals, known for their rapid reaction yet fleeting presence in aqueous environments, pose detection challenges; in contrast, superoxide anions, generated through electron acceptance by oxygen molecules in electron transfer processes, participate widely in biological reactions and exhibit greater stability in water. Consequently, the experiment primarily detected superoxide anion signals. Figure 7 clearly shows that the magnetic field intensifies superoxide anion detection. Numerous studies have indicated that biological responses to magnetic fields often involves oxidative stress, defined as a shift towards oxidants in the oxidant–antioxidant balance [35]. Calabrò et al. [36] reported that the exposure to static magnetic fields of 2.2 mT increased the intracellular ROS levels in human neuroblastoma cells, while an induction of ROS in cardiac progenitor cells derived from mouse cells exposed to a 0.2–5 mT static magnetic field was reported by Bekhite Mohamed et al. [37]. These phenomena could be elucidated by the radical pair mechanism, wherein magnetic fields influenced the electron spin, thereby extending the duration necessary for free radical recombination and consequently prolonging their effective lifetime [38]. Furthermore, the superoxide anion could accept an H^+^ to form the protonated superoxide anion radical, indicating its basic nature in water [39]. The enhanced concentration of superoxide anions, coupled with changed water cluster properties, may explain the improved efficacy of pH-shifting treatment in the presence of magnetic field; the schematic diagram is depicted in Figure 8.

## 4. Conclusions

In industrial production, research into pH-shifting treatments has made progress in improving the quality of low-value proteins from aquatic and poultry by-products. Due to its simple structure, magnetic field equipment is often combined with other methods to improve processing effects. Examples include commercialized magnetic induction heat reactors, magnetic induction electric field hydrolysis extraction systems, and magnetic field freezing and thawing units. Combining pH-shifting technology with magnetic field technology is feasible for implementation in industrial productions. And the present results show that the magnetic field-assisted pH-shifting treatments could be an effective method to modify the protein properties of the frozen meat. In the pH-shifting process, an increase in pH enhanced the protein solubility and altered the structure conformations. Nevertheless, excessively high pH levels induced covalent aggregation of the proteins. This was attributed to the increased reactivity of thiol groups under alkaline conditions and the formation of non-disulfide covalent bonds within the MPs in such environments. Combining a magnetic field into the pH-shifting process effectively reduced the required pH values, accelerating the protein structural modifications and inter-protein interactions. The improving effects may be associated with the increased free radical concentration and the property changes in the water clusters due to the magnetic field. Amino acid analysis indicated that neither the pH-shifting treatment nor the magnetic field-assisted pH-shifting treatments significantly changed the amino acid profile, implying minimal impact on the nutritional quality of frozen meat.

## Figures and Tables

**Table 1:** The effect of different pH-shifting treatments on the thermal denaturation temperature and enthalpy (ΔH, mJ/g) of MP.

Samples	T_1_ (°C)	T_2_ (°C)	ΔH_1_ (mJ/g)	ΔH_2_ (mJ/g)
MP	50.34 ± 0.59 ^c^	69.11 ± 0.41 ^e^	76.27 ± 3.03 ^f^	26.49 ± 1.53 ^d^
MP-9	49.51 ± 0.67 ^bc^	66.28 ± 0.54 ^d^	68.89 ± 2.52 ^de^	24.23 ± 1.32 ^cd^
MP-10	49.03 ± 0.12 ^ab^	64.80 ± 0.21 ^c^	60.07 ± 2.33 ^bc^	22.30 ± 1.65 ^bc^
MP-11	48.88 ± 0.14 ^ab^	63.74 ± 0.11 ^b^	55.83 ± 2.23 ^ab^	19.98 ± 1.06 ^ab^
MP-9+	49.34 ± 0.06 ^abc^	65.94 ± 0.16 ^d^	70.19± 2.62 ^ef^	23.78 ± 0.82 ^cd^
MP-10+	49.25 ± 0.43 ^abc^	64.71 ± 0.55 ^bc^	62.85 ± 1.79 ^cd^	22.78 ± 1.56 ^bcd^
MP-11+	48.30± 0.22 ^a^	62.37± 0.12 ^a^	53.35± 2.02 ^a^	17.45 ± 1.27 ^a^

Note: Values were presented as the mean ± standard deviation of three replicates. Different superscripts (a–f) in the same column denoted significant differences (*p* < 0.05, *n* = 3) among the various treatments. MP represents the untreated sample. MP-9, MP-10, MP-11 represent the samples of pH-shifting treatments to pH 9, pH 10, pH 11. MP-9+, MP-10+, and MP-11+ represent the samples subjected to magnetic field-assisted pH-shifting treatments, achieving pH levels of 9, 10, and 11, respectively.

**Table 2:** The effect of different pH-shifting treatments on the amino acid contents of MP.

Samples (%)	MP	MP-9	MP-10	MP-11	MP-9+	MP-10+	MP-11+
Aspartate	9.98 ± 0.03 ^d^	9.63 ± 0.08 ^b^	9.78 ± 0.07 ^c^	10.2 ± 0.05 ^d^	9.88 ± 0.04 ^e^	9.88 ± 0.02 ^cd^	9.49 ± 0.03 ^a^
Threonine	6.31 ± 0.05 ^d^	6.08 ± 0.09 ^ab^	6 ± 0.06 ^a^	6.13 ± 0.02 ^abc^	6.13 ± 0.03 ^abc^	6.27 ± 0.06 ^cd^	6.2 ± 0.04 ^bcd^
Serine	4.97 ± 0.05 ^b^	4.84 ± 0.10 ^ab^	4.9 ± 0.09 ^ab^	4.99 ± 0.03 ^b^	4.93 ± 0.02 ^ab^	4.81 ± 0.04 ^a^	4.88 ± 0.03 ^ab^
Glutamic acid	15.39 ± 0.11 ^cd^	15.1 ± 0.03 ^ab^	15.23 ± 0.09 ^bc^	15.46 ± 0.06 ^d^	15.25 ± 0.05 ^bc^	15.04 ± 0.04 ^a^	15.32 ± 0.03 ^cd^
Glycine	3.6 ± 0.03 ^ab^	3.67 ± 0.02 ^b^	3.68 ± 0.07 ^b^	3.51 ± 0.04 ^a^	3.58 ± 0.08 ^ab^	3.53 ± 0.03 ^a^	3.53 ± 0.04 ^a^
Alanine	5.38 ± 0.04 ^cd^	5.15 ± 0.07 ^ab^	5.17 ± 0.05 ^ab^	5.27 ± 0.03 ^bc^	5.24 ± 0.06 ^bc^	5.07 ± 0.07 ^a^	5.42 ± 0.04 ^d^
Cystine	0.79 ± 0.05 ^c^	0.72 ± 0.04 ^bc^	0.68 ± 0.03 ^ab^	0.71 ± 0.02 ^abc^	0.65 ± 0.03 ^ab^	0.70 ± 0.05 ^abc^	0.61 ± 0.04 ^a^
Valine	4.51 ± 0.05 ^bcd^	4.35 ± 0.03 ^a^	4.52 ± 0.04 ^cd^	4.53 ± 0.04 ^cd^	4.58 ± 0.04 ^d^	4.45 ± 0.04 ^abc^	4.39 ± 0.05 ^ab^
Methionine	4.09 ± 0.07 ^bc^	4.21 ± 0.04 ^c^	3.96 ± 0.08 ^ab^	3.92 ± 0.03 ^a^	4.05 ± 0.03 ^ab^	3.99 ± 0.02 ^ab^	3.94 ± 0.04 ^a^
Isoleucine	6.49 ± 0.05 ^a^	7.83 ± 0.04 ^d^	6.75 ± 0.05 ^b^	6.66 ± 0.03 ^b^	6.66 ± 0.04 ^b^	6.91 ± 0.05 ^c^	6.77 ± 0.01 ^c^
Proline	1.19 ± 0.01 ^d^	0.96 ± 0.03 ^ab^	1.08 ± 0.02 ^c^	0.88 ± 0.06 ^a^	0.93 ± 0.04 ^ab^	1.02 ± 0.01 ^bc^	0.86 ± 0.05 ^a^
Leucine	8.49 ± 0.09 ^a^	8.82 ± 0.08 ^c^	8.66 ± 0.07 ^bc^	8.54 ± 0.04 ^b^	8.48 ± 0.03 ^a^	8.73 ± 0.03 ^c^	8.7 ± 0.04 ^bc^
Tyrosine	5.01 ± 0.05 ^a^	5.04 ± 0.06 ^ab^	5.00 ± 0.04 ^a^	5.01 ± 0.05 ^ab^	5.03 ± 0.04 ^ab^	5.11 ± 0.06 ^ab^	5.14 ± 0.03 ^b^
Phenylalanine	4.84 ± 0.09 ^a^	4.75 ± 0.04 ^a^	5.01 ± 0.05 ^b^	5.02 ± 0.07 ^b^	5.03 ± 0.03 ^b^	5.02 ± 0.06 ^b^	5.05 ± 0.03 ^b^
Histidine	3.37 ± 0.04 ^ab^	3.27 ± 0.02 ^a^	3.37 ± 0.05 ^ab^	3.34 ± 0.03 ^ab^	3.33 ± 0.03 ^ab^	3.29 ± 0.05 ^a^	3.42 ± 0.04 ^b^
Lysine	9.31 ± 0.04 ^a^	9.34 ± 0.03 ^a^	9.54 ± 0.05 ^c^	9.46 ± 0.04 ^bc^	9.7 ± 0.03 ^d^	9.54 ± 0.03 ^c^	9.41 ± 0.05 ^ab^
Arginine	7.44 ± 0.05 ^a^	7.18 ± 0.08 ^a^	7.76 ± 0.04 ^c^	7.27 ± 0.05 ^a^	7.48 ± 0.04 ^b^	7.67 ± 0.05 ^c^	7.64 ± 0.05 ^c^

Note: Each experiment was repeated three times, and the data were expressed as mean ± standard deviation. There were significant differences among different letters in the same row (*p* < 0.05). MP represents the untreated sample. MP-9, MP-10, MP-11 represent the samples of pH-shifting treatments to pH 9, pH 10, pH 11. MP-9+, MP-10+, and MP-11+ represent the samples subjected to magnetic field-assisted pH-shifting treatments, achieving pH levels of 9, 10, and 11, respectively.

## Data Availability

The original contributions presented in the study are included in the article, further inquiries can be directed to the corresponding author.
